# Design of the Japan Kidney Association-Pemafibrate Intervention for Chronic Kidney Disease patients Study (JKAPI-CKD Study)

**DOI:** 10.1093/ckj/sfag053

**Published:** 2026-02-23

**Authors:** Sho Kinguchi, Kouichi Tamura, Yuichiro Yano, Masaomi Nangaku, Yoshitaka Isaka, Shoichi Maruyama, Hiroshi Kanegae, Hiddo Lambers Heerspink, Naoki Nakagawa, Saori Nishio, Koichi Asahi, Kunihiro Yamagata, Akira Fukui, Hirokazu Okada, Ichiei Narita, Kazuhiko Tsuruya, Jun Wada, Yoshio Terada, Masashi Mukoyama, Enyu Imai, Naoto Yokota, Kazuo Kobayashi, Naoki Kashihara

**Affiliations:** Department of Medical Science and Cardiorenal Medicine, Yokohama City University Graduate School of Medicine, Yokohama, Japan; Department of Nephrology and Hypertension, Yokohama City University Medical Center, Yokohama, Japan; Department of Medical Science and Cardiorenal Medicine, Yokohama City University Graduate School of Medicine, Yokohama, Japan; Department of Nephrology and Hypertension, Yokohama City University Medical Center, Yokohama, Japan; Department of General Medicine, Juntendo University Faculty of Medicine, Hongo, Bunkyo-ku, Tokyo, 113-8421, Japan; Division of Nephrology and Endocrinology, The University of Tokyo Graduate School of Medicine, Hongo, Bunkyo-ku, Tokyo, 113-0033, Japan; Department of Nephrology, The University of Osaka Graduate School of Medicine, Suita, Japan; Department of Nephrology, Nagoya University Graduate School of Medicine, Nagoya, Japan; Genki Plaza Medical Centre for Health Care, Chiyoda-ku, Tokyo, 101-0051, Japan; Department of Clinical Pharmacy and Pharmacology, University Medical Center Groningen, University of Groningen, Groningen, 9713 GZ, the Netherlands; Division of Cardiology and Nephrology, Department of Internal Medicine, Asahikawa Medical University, Hokkaido, 078-8510, Japan; Internal Medicine II, Hokkaido University Hospital, Sapporo, Japan; Division of Nephrology and Hypertension, Department of Internal Medicine, Iwate Medical University School of Medicine, Yahaba, Japan; Department of Nephrology, Faculty of Medicine, University of Tsukuba, Tsukuba, Japan; Division of Nephrology and Hypertension, Department of Internal Medicine, The Jikei University School of Medicine, Minato-ku, Tokyo, 105-8461, Japan; Department of Nephrology, Saitama Medical University, Saitama, Japan; Niigata Prefecture Health Promotion and Sports Medical Science Center, Niigata Prefecture Sports Association, Niigata, 950-0933, Japan; Department of Nephrology, Nara Medical University, Nara, 634-8522, Japan; Department of Nephrology, Rheumatology, Endocrinology and Metabolism, Okayama University Faculty of Medicine, Dentistry and Pharmaceutical Sciences, Okayama, 700-8558, Japan; Department of Endocrinology, Metabolism & Nephrology, Kochi Medical School, Kochi University, Kochi, 783-0043, Japan; Department of Nephrology, Omuta Tenryo Hospital, Fukuoka, 836-8566, Japan; Nakayamadera Imai Clinic, Hyogo, 665-0861, Japan; Yokota Naika, Miyazaki, 880-0036, Japan; Kobayashi Internal Medicine Clinic, Kanagawa, 229-1131, Japan; Kawasaki Geriatric Medical Center, Okayama, 700-0821, Japan

**Keywords:** chronic kidney disease, eGFR slope, hypertriglyceridemia, pemafibrate, selective PPARα modulator

## Abstract

**Background:**

Hypertriglyceridemia has attracted considerable attention as a residual cardiovascular risk factor in patients with chronic kidney disease (CKD). Pemafibrate, a selective peroxisome proliferator-activated receptor α modulator, is a novel therapeutic option for hypertriglyceridemia in patients with CKD, and is associated with fewer kidney adverse events in this population compared with conventional fibrates. Although appropriate management of hypertriglyceridemia may contribute to improved kidney outcomes, evidence regarding the efficacy and safety of pemafibrate to reduce CKD progression is unknown. The aim of this study is to investigate the effect of pemafibrate on the rate of change in estimated glomerular filtration rate (eGFR) slope in patients with CKD.

**Methods:**

The Japan Kidney Association-Pemafibrate Intervention for Chronic Kidney Disease patients (JKAPI-CKD) study is a prospective, multicenter, open-label, parallel-group, randomized controlled trial. The study will enroll 2200 adults with CKD, defined as eGFR ≥20 ml/min/1.73 m^2^ and <60 ml/min/1.73 m^2^, and hypertriglyceridemia [triglyceride level ≥150 mg/dl (fasting) or ≥175 mg/dl (non-fasting)] who have not received pemafibrate or conventional fibrates within 12 weeks prior to consent. Participants will be randomized in a 1:1 ratio to the pemafibrate group (guideline-based conventional therapy plus pemafibrate 0.1–0.4 mg/day) or the control group (guideline-based conventional therapy excluding fibrates). The observation period will be 104 weeks. The primary endpoint is the chronic eGFR slope, defined as the eGFR slope from weeks 12 to 104 after study initiation.

**Results:**

On 1 December 2025, a total of 2328 participants had been enrolled, completing the participant enrollment.

**Conclusions:**

The JKAPI-CKD study is anticipated to yield novel insights into the renoprotective effects of pemafibrate in patients with CKD and establish new evidence supporting its use for the management of dyslipidemia in this population.

Trial registration: Japan Registry of Clinical Trials (jRCTs031240097)

## Graphical Abstract

**Graphical Abstract:**
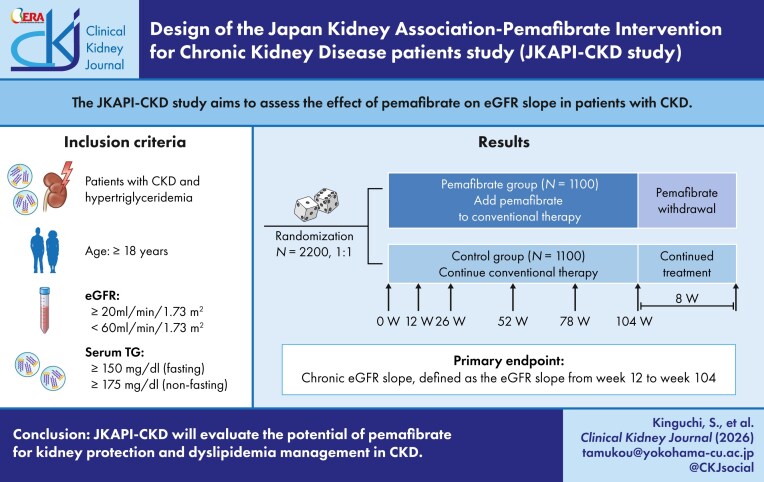

KEY LEARNING POINTS **What was known**:

- Patients with chronic kidney disease (CKD) have altered lipid profiles (high triglyceride (TG) levels, low high-density lipoprotein cholesterol (HDL-C) levels, and elevated TG/HDL-C ratio), which are associated with an increased risk of cardio-kidney events.
- Pemafibrate safely improves lipid profiles in patients with CKD.

**This study adds**:

- JKAPI-CKD is a randomized controlled trial designed to assess the effect of pemafibrate on the estimated glomerular filtration rate slope in patients with CKD.
- JKAPI-CKD has a large sample size (*N* = 2200) and a long follow-up period (104 weeks), enabling various analyses that may yield clinically meaningful findings.

**Potential impact**:

- JKAPI-CKD will yield novel insights into the renoprotective effects of pemafibrate in patients with CKD and establish new evidence supporting its use for the management of dyslipidemia in this population.

## INTRODUCTION

Patients with chronic kidney disease (CKD) are at high risk of kidney failure, cardiovascular events (CVE), and mortality [1]. Regarding treatment of dyslipidemia in patients with CKD, low-density lipoprotein cholesterol (LDL-C) lowering therapy with statins is recommended [2, 3]. Recently, hypertriglyceridemia has emerged as a novel risk factor and potential therapeutic target for reducing the risks of end-stage kidney disease (ESKD), CV outcomes, and mortality. A meta-analysis demonstrated that triglyceride (TG)-lowering therapy is associated with a reduced risk of CV outcomes and mortality rates in patients with type 2 diabetes [4]. In a randomized controlled trial (RCT) (the FIELD trial), fenofibrate reduced the decline in eGFR in patients with type 2 diabetes [5]. In another US-based national cohort study, fibrate use was associated with a lower risk of ESKD and mortality in veterans [6].

Previous large cohort studies showed that CKD patients were prone to altered lipid profiles [high TG level, low high-density lipoprotein cholesterol (HDL-C) level, and high TG/HDL-C ratio], which were associated with the incidence and progression of CKD [7–10]. In the ARIC study, hypertriglyceridemia was also a risk factor for coronary artery disease in CKD patients [11]. However, because conventional fibrates are a risk factor for kidney damage, their use in CKD patients is not recommended and is contraindicated, especially in patients with severe CKD [12]. Thus, evidence for fibrate use in CKD patients is limited.

Pemafibrate is a selective peroxisome proliferator-activated receptor alpha modulator, which lowers TG and increases HDL-C [13]. Because pemafibrate is excreted primarily in the bile, unlike conventional fibrates, its use is approved in patients with severe CKD [14]. A *post hoc* analysis of a phase III trial demonstrated that pemafibrate improved hypertriglyceridemia without significantly worsening kidney function, regardless of baseline CKD stages [15]. In a phase IV trial, no significant changes in pemafibrate pharmacokinetics were observed in patients with severe CKD compared with patients with mild-to-moderate CKD [14]. In some small observational studies in patients with CKD, switching from conventional fibrates to pemafibrate maintained a similar TG-lowering effect and led to a decrease in serum creatinine (Cr) level and an increase in estimated glomerular filtration rate (eGFR) [16, 17]. Furthermore, a large nested case-control study demonstrated that pemafibrate use, but not conventional fibrate use, was significantly associated with a decreased risk of major adverse cardiovascular events (MACE) in Japanese CKD patients [18]. Therefore, pemafibrate can be used safely in CKD patients and could slow the progression of CKD and reduce the risk of CV outcomes. However, adequately designed and well powered RCTs examining the efficacy and safety of pemafibrate in patients with CKD have not been initiated. The evidence for pemafibrate in managing dyslipidemia in patients with CKD is therefore unknown.

To address this knowledge gap, the Japan Kidney Association-Pemafibrate Intervention for Chronic Kidney Disease (JKAPI-CKD) patients Study has been designed to examine the effect of pemafibrate on kidney function (eGFR slope) in patients with hypertriglyceridemia and non-dialysis dependent CKD.

## MATERIALS AND METHODS

### Study design and study organization

**Figure 1:**
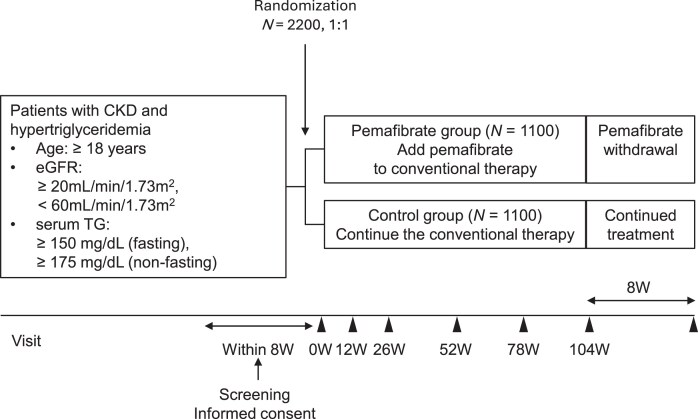
Study design. Participants are patients with hypertriglyceridemia complicated by CKD. In the pemafibrate group, pemafibrate will be added to conventional treatment. In the control group, conventional treatment will be continued throughout the study period. In both groups, treatment will be ongoing in accordance with clinical guidelines, excluding fibrates. Participant assessment will begin within 8 weeks of obtaining informed consent, and the treatment period will last 104 weeks. After completion of the 104-week treatment period, participants in the pemafibrate group will discontinue pemafibrate, continue conventional treatment, and will be evaluated 8 weeks later. Participants in the control group will continue conventional treatment and will be evaluated 8 weeks after the 104-week visit. W, weeks.

The JKAPI-CKD study is a prospective, multicenter, open-label, parallel-group RCT (Fig. 1). Participant recruitment started in May 2024 and will conclude in June 2026. The scheduled study period is from May 2024 to June 2029. This study has been registered with the Japan Registry of Clinical Trials (jRCTs031240097) and is led by the Japan Kidney Association (JKA). The Kowa Company, Ltd, that manufactures pemafibrate, has provided funding for the JKAPI-CKD study through a grant to the JKA. The Steering Committee’s responsibilities comprise the preparation of the protocol, supervision of the clinical trial, review of the information and clinical trial status of each facility, and resolving issues during the study (Supplementary File 1). A biostatistician (Director of Biostatistics) and a Clinical Design Adviser will provide advice regarding the protocol and statistical analysis plan. The monitoring organization appointed by the principal investigator will regularly monitor study progress and compliance with the plan and ethical guidelines. The participating medical institutions as of 1 August 2025 are listed in Supplementary File 1. The study was approved by the Certified Review Board of Yokohama City University (CRB3180007) and will be performed in accordance with the Declaration of Helsinki and Clinical Trials Act. Written informed consent will be required from all participants before enrollment.

### Eligibility

The JKAPI-CKD study will recruit 2200 participants with hypertriglyceridemia complicated by CKD at 497 medical institutions in Japan. Adults ≥18 years of age are eligible. Participants are also required to have CKD, determined using the most recent laboratory results from samples obtained within 16 weeks prior to consent, with CKD defined as eGFR ≥20 and <60 ml/min/1.73 m². Hypertriglyceridemia is defined as fasting serum TG level ≥150 mg/dl or non-fasting serum TG level ≥175 mg/dl at least once within 16 weeks prior to consent. Table 1 shows the eligibility criteria. The major exclusion criteria comprise the use of a fibrate or pemafibrate within 12 weeks prior to consent; pemafibrate contraindications; severe proteinuria [urinary protein-to-creatinine ratio (UPCR) >5 g/g Cr or urinary albumin-to-creatinine ratio (UACR) >3000 mg/g Cr]; severe hypertriglyceridemia (serum TG >500 mg/dl); and high creatine kinase level (>5× the upper limit of normal) using the latest measurement within 16 weeks prior to consent. Patients with severe kidney insufficiency (eGFR <20 ml/min/1.73 m^2^ within 16 weeks prior to consent), undergoing dialysis, or who have a history of kidney transplantation are also excluded (Table 1).

**Table 1:** Inclusion and exclusion criteria.

**Inclusion criteria**
1. Age 18 years or older at informed consent
2. Patients with CKD; eGFR^a^ ≥20 ml/min/1.73 m^2^, <60 ml/min/1.73 m^2^
3. Serum TG ≥150 mg/dl (fasting), or ≥175 mg/dl (non-fasting), at least once within 16 weeks prior to consent
4. No changes in dyslipidemic medications, antihypertensive medications, or antidiabetic medications within 12 weeks prior to consent
5. Written informed consent for study enrollment obtained from the patient
**Exclusion criteria**
1. Use of fibrate or pemafibrate within 12 weeks prior to consent
2. Contraindications of pemafibrate based on the latest drug information
2–1. History of hypersensitivity to pemafibrate
2–2. Severe liver dysfunction, cirrhosis Child–Pugh class B or C, or biliary obstruction
2–3. Gallstones
2–4. Women who are pregnant, or lactating
2–5. Taking cyclosporine, or rifampin
3. UPCR >5 g/gCr, or UACR >3000 mg/gCr^a^
4. Serum TG >500 mg/dl^a^
5. Uncontrolled high blood pressure defined as SBP ≥180 mmHg, or DBP ≥110 mmHg^a^
6. HbA1c ≥8.5%^a^
7. CK > 5× ULN^a^
8. eGFR <20 ml/min/1.73 m^2^ within 16 weeks prior to consent, undergoing dialysis, or history of kidney transplantation
9. Under treatment for malignancy (except for intraepithelial neoplasia)
10. Participants in other interventional studies and clinical trials
11. Patients deemed ineligible by the investigators

a Using the latest measurement in the 16 weeks prior to consent. CK, creatine kinase; DBP, diastolic blood pressure; HbA1c: hemoglobin A1c; ULN, upper limit of normal.

### Registration and randomization

Investigators will obtain written consent from the participants after confirming that the eligibility criteria are met. This study will use an online electronic data capture system (Viedoc™ version 4 (Viedoc4); Viedoc Technologies AB, Uppsala, Sweden). Investigators will register participants using the Viedoc4 web registration system. Viedoc4 assigns a research number and treatment group for each participant. Eligible participants will be randomly allocated to the pemafibrate or control groups in a 1:1 ratio. Age (<65 years vs. ≥65 years), eGFR (<45 ml/min/1.73 m^2^ vs. ≥45 ml/min/1.73 m^2^), presence/absence of diabetes, and presence/absence of hypertension are the stratification factors because these are involved in the extent of eGFR changes.

### Treatment

#### Study treatment

Pemafibrate (immediate-release tablet or extended-release tablet) will be administered orally in the pemafibrate group within 8 weeks after enrollment. Patients in the pemafibrate group will receive pemafibrate 0.1–0.4 mg daily for 104 weeks in accordance with the latest instructions on the package insert. The pemafibrate dosage will be adjusted to achieve the TG target (serum TG level <150 mg/dl (fasting) or <175 mg/dl (non-fasting)). The maximum pemafibrate dose is 0.4 mg daily; dose reduction may be considered in patients with hepatic or kidney dysfunction. The maximum pemafibrate dose is 0.2 mg daily for patients with eGFR <30 ml/min/1.73 m^2^. In the control group, conventional treatment will be continued, with observation initiated within 8 weeks after enrollment. With rapid exacerbation of kidney function (i.e. increase in serum creatinine to ≥1.5 times the baseline value or decline in eGFR of ≥40% from baseline), marked increase in creatine kinase (≥5× the upper limit of normal), or myoglobinuria (reddish-brown urine) during the study period, pemafibrate dose reduction or discontinuation will be considered in the pemafibrate group. Investigators are advised to follow local or international practice guidelines to optimize kidney protective treatment.

#### Concomitant therapy

Treatment in accordance with relevant guidelines [2, 19] will continue for 104 weeks after enrollment in both groups. Fibrates cannot be used during the study period. In principle, change or dose adjustment of dyslipidemia medications other than pemafibrate will not be permitted during the study period. However, when an investigator decides that a change in dyslipidemia medication is unavoidable, the change must be recorded in Viedoc4. When concomitant medications that may affect kidney function, such as medications for kidney impairment, hypertension, or diabetes, or urate-lowering drugs, are also changed, the change must be recorded in Viedoc4.

#### Follow up

The study schedule is summarized in Supplementary Table 1. Follow-up visits are scheduled at 12 and 26 weeks and then every 26 weeks until 104 weeks. After 104 weeks of observation, participants in the pemafibrate group will stop taking pemafibrate and continue conventional therapy, and will be assessed 8 weeks after discontinuation. Participants in the control group will continue conventional treatment and will be assessed 8 weeks after the 104-week visit. Adherence to study treatment will be assessed by asking participants about missed doses. Blood pressure, pulse rate, and body weight measurements will be obtained at each visit. Blood and urine laboratory tests will also be performed at each visit. Laboratory parameters will be assessed using routine methods in participating medical facilities. In principle, fasting blood samples will be collected (≥10 hours after the last meal). If non-fasting blood samples are collected at baseline, non-fasting blood samples will also be collected during follow up. Details of the laboratory measurements are shown in Supplementary Table 2. LDL-C (direct method) and cystatin C will also be measured if possible (i.e. with insurance coverage). LDL-C (calculated), non-HDL-C, TG/HDL-C ratio, small dense LDL-C, TG-rich lipoprotein cholesterol, eGFR, and eGFR based on cystatin C (eGFR-cys) will be calculated using the formulas described in the Supplementary Methods (Supplementary File 2). Urinalysis will be performed on spot urine samples to assess UACR, if possible (i.e. with insurance coverage), or UPCR. At each visit, concomitant medications, cardio-kidney events, and adverse events (AEs) will be verified by the investigators.

#### Endpoints

The primary endpoint of the JKAPI-CKD study is the chronic eGFR slope from 12 to 104 weeks, thereby excluding potential acute changes in eGFR. Chronic eGFR slope is a proposed surrogate marker for kidney events [20, 21]. The key secondary endpoint is total eGFR slope calculated from randomization until week 104. Other secondary and safety endpoints are shown in Table 2. In the present study, some pre-specified subgroup analyses are planned (Supplementary Table 3), including analyses of the stratification factors used in the randomization process (baseline eGFR category, presence of diabetes, and presence of hypertension). Outcomes in the subgroups will be examined to test for interaction effects.

**Table 2:** Outcomes.

Primary endpoint
Chronic eGFR slope from 12 to 104 weeks, after study start
**Secondary endpoints**
Key secondary endpoint
Total eGFR slope during the 104-week study period
Efficacy endpoints
Change from baseline in the following items at each visit
Lipid metabolism^a^
TG, HDL-C, TC, LDL-C, non-HDL-C, TG/HDL-C, and TRL-C
Kidney function
eGFR, Cr, UPCR, UACR, and predicted ACR^b^
Change in proportion of CKD stages between baseline and 104-week visit
Proportion of CKD stage transition between baseline and 104-week visit
Liver function
AST, ALT, γ-GTP, and ALP
Others
CK, BUN, uric acid, total protein, albumin, CRP, RBC, WBC, blood glucose^a^, Hb, Hct, platelet count, HbA1c (diabetic patients only), BNP (NT-proBNP) (heart failure patients only)
Outcomes
Incidence of composite kidney events, and components of kidney events
Incidence of composite CVEs, and components of CVEs
Incidence of rhabdomyolysis
Incidence of acute kidney injury
Physical findings
Body weight, body mass index, blood pressure (rest sitting position), pulse rate
Exploratory items
eGFR-cys, and cystatin C
Comparison of eGFR slope before the study and during the study^c^
Change from baseline in the above items at each visit and 8 weeks after 104-week visit in the cases with data available
Correlation between change in TG and other variables
Safety evaluation
Incidence of severe AEs
Incidence of AEs
Incidence of severe side-effects related to the study drug
Incidence of side-effects related to the study drug

a Lipid metabolism-related items and blood glucose analyzed separately for fasting and non-fasting blood samples.

b predicted ACR = exp 5.2659 + 0.2934 × log [min (PCR/50, 1)] + 1.5643 × log {max [min (PCR/500, 1), 0.1]} + 1.1109 × log [max (PCR/500, 1)] − 0.0773 × (if female) + 0.0797 × (if diabetic) + 0.1265 × (if hypertensive).

c eGFR slope during the study includes both chronic and total eGFR slopes. ACR, albumin-to-creatinine ratio; ALP, alkaline phosphatase; ALT, alanine aminotransferase; AST, aspartate aminotransferase; BNP, brain natriuretic peptide; BUN, blood urea nitrogen; CK, creatine kinase; Cr, creatinine; CRP, C-reactive protein; CVE, cardiovascular event; eGFR-cys, eGFR based on cystatin C; γ-GTP, gamma-glutamyl transpeptidase; Hb, hemoglobin; HbA1c, hemoglobin A1c; Hct, hematocrit; HDL-C, high-density lipoprotein cholesterol; LDL-C, low-density lipoprotein cholesterol; NT-proBNP, N-terminal pro BNP; RBC, red blood cell count; TC, total cholesterol; TRL-C, TG-rich lipoprotein cholesterol; WBC, white blood cell count.

### Statistical analysis

#### Sample size

On the basis of a previous study [20] and a guideline [21], a difference of 0.5–1.0 ml/min/1.73 m^2^/year in eGFR slope has been shown to be associated with a >97.5% probability that the drug will also reduce the risk of kidney failure. Therefore, we defined a difference of 0.5 ml/min/1.73 m^2^/year in eGFR slope between the study groups as a non-inferiority margin, assuming that the standard deviation (SD) would be 4.0 [22]. This SD estimate was based on a previous study of Japanese patients with type 2 diabetes and renal dysfunction [22]. This estimate was considered appropriate because it was derived from clinic-based real-world data in Japan, which closely reflects the multicenter clinical setting and patient characteristics of the present study. Moreover, because the JKAPI-CKD study applies rigorous inclusion and exclusion criteria (e.g. eGFR ≥20 and <60 ml/min/1.73 m² and exclusion of severe proteinuria), the study population is expected to be more homogeneous than general real-world cohorts, further supporting the validity of this assumption. The target enrollment of 2200 participants was calculated to detect a difference in the primary endpoint of 0.5 ml/min/1.73 m^2^/year eGFR slope difference with a power of 80% and an alpha error of 2.5%, accounting for a potential 10% drop-out rate.

#### Analysis set

Three analysis populations are defined for this trial: (i) the full analysis set (FAS), defined as all eligible participants who received at least one dose of the study treatment and had at least one observation in the protocol therapy period for the analysis endpoint; (ii) the per-protocol set, defined as participants in the FAS, without significant violation of the study protocol; and (iii) the safety analysis set, defined as participants who received the study drug at least once. The main efficacy analyses will be performed using the FAS population data.

#### Efficacy analysis

The annual rate of change in eGFR from 12 to 104 weeks (chronic eGFR slope) will be analyzed using a random coefficient model. In this model, fixed effects comprise the treatment group, observation time point, and the interaction between the treatment group and observation time point; random effects comprise the intercept and observation time point. Chronic eGFR slope will be compared between the treatment and control groups in the following order: (i) the non-inferiority of the pemafibrate group to the control group will be examined. Non-inferiority will be concluded if the lower limit of the 95% confidence interval (CI) for the eGFR slope in the pemafibrate group compared with the control group is >−0.5. (ii) The superiority of the pemafibrate group will be examined if non-inferiority is achieved. The difference will be considered statistically significant when the lower limit of the 95% CI for the eGFR slope in the pemafibrate group compared with the control group is >0.0. Randomization will be stratified by age, baseline eGFR category, diabetes status, and hypertension status. These stratification factors will be adjusted as covariates in the primary analysis model because they are established predictors of eGFR decline and are expected to reduce residual variance.

The annual rate of change in eGFR during the 104-week study period (total eGFR slope) will be analyzed using the same procedure as that for chronic eGFR slope. Regarding changes in other variables, the least-squares mean, and its 95% CI for each visit will be calculated using the mixed-effects models for repeated measures, and differences between the groups will be calculated. Comparisons between the groups will be performed using the unpaired *t*-test. The proportions of participants in each CKD stage and grade of urinary protein qualitative evaluation will be evaluated using the Chi-square test. Regarding the outcome measures, the incidence rates will be calculated using the person-year method, and survival curves will be confirmed using the Kaplan–Meier method. When adjusting for covariates, a Cox proportional hazards model will be used to calculate hazard ratios. Stratified analyses will be performed for the endpoints, such as by fasting or non-fasting status, medical history, or concomitant medications. Sensitivity analysis will also be performed to adjust for potential confounding factors, such as sex or UACR at enrollment.

Given that the primary endpoint is based on eGFR, additional sensitivity analyses will be performed. These analyses will exclude participants with conditions that potentially bias eGFR estimation, such as severe obesity (e.g. body mass index ≥35 kg/m²), nephrotic-range proteinuria (e.g. UPCR ≥3.5 to ≤5.0 g/g Cr), or marked malnutrition (e.g. body mass index <18 kg/m² or albumin <3.0 g/dl), to assess the robustness of the primary findings.

### Safety analysis

The safety analysis will be performed using data for the safety analysis set population. AEs during the study period will be summarized using frequency counts and percentages, with 95% CIs calculated by the Clopper–Pearson method. Serious AEs and treatment-related AEs will be summarized similarly. All AEs will be graded for severity in accordance with the Common Terminology Criteria for Adverse Events, version 5.0.

## RESULTS

On 1 December 2025, a total of 2328 participants had been enrolled, completing the participant enrollment.

## DISCUSSION

The JKAPI-CKD study is, to the best of our knowledge, the first randomized controlled clinical trial to rigorously evaluate the effect of pemafibrate on eGFR slope in patients with hypertriglyceridemia and CKD.

A large Japanese cohort study reported that an elevated TG/HDL-C ratio was associated with the incidence and progression of CKD [7, 8]. Furthermore, the frequency of hypertriglyceridemia and hypo-HDL-cholesterolemia increases as CKD progresses [9]. A large, community-based, longitudinal study showed that CKD was associated with a higher risk of new-onset high-TG, low-HDL-C, and high-TG/HDL-C ratio compared with non-CKD patients [10]. However, previous guidelines did not recommend the use of fibrates in CKD patients because conventional fibrates may worsen kidney damage [12]. Evidence for the use of conventional fibrates in patients with severe CKD is limited because these medications are contraindicated in patients with severe CKD. A meta-analysis of 10 RCTs assessed the effects of fibrate therapy compared with placebo in people with CKD or on kidney-related outcomes [23]. The study showed that fibrates increased serum creatinine levels and reduced eGFR; however, lipid profiles improved (i.e. lowered TG levels and increased HDL-C levels), and the risk of CV outcomes decreased in patients with mild-to-moderate CKD [23].

Pemafibrate is a novel selective peroxisome proliferator-activated receptor alpha modulator that improves lipid profiles and is excreted primarily in the bile [13]. Unlike conventional fibrates, pemafibrate can be used safely in patients with severe CKD. In a phase IV study, no significant changes in the pharmacokinetics of pemafibrate were observed in patients with severe CKD (eGFR <30 ml/min/1.73 m^2^) or in those receiving maintenance dialysis compared with patients with mild-to-moderate CKD (i.e. eGFR ≥30 and <60 ml/min/1.73 m^2^) [14]. *Post hoc* analyses by subgroups stratified on the basis of baseline eGFR showed that pemafibrate exerted TG-lowering effects and did not worsen kidney function regardless of baseline CKD stage during 52 weeks of follow up in a multicenter, single-arm, open-label, phase III trial [15]. However, in the PROMINENT trial, which was a large RCT assessing the efficacy of pemafibrate on CV outcomes in patients with type 2 diabetes, increased serum creatinine levels and decreased eGFR were observed during pemafibrate administration [24]. Notably, these changes were reversible after discontinuation of pemafibrate. The changes in serum creatinine and eGFR observed with pemafibrate may, at least in part, reflect its effects on glomerular hemodynamics [25–27] because patients with diabetes often develop glomerular hyperfiltration. Some small observational studies reported that switching from conventional hypertriglyceridemia treatment, including fibrates, to pemafibrate did not lead to an increase in serum creatinine or a decline in eGFR, in patients with CKD, not limited to those with diabetic nephropathy [16, 17]. Notably, switching from conventional fibrates to pemafibrate was associated with improved kidney function, as evidenced by reduced serum creatinine levels and increased eGFR [16, 17]. An RCT is ongoing to examine the effect of pemafibrate on proteinuria in patients with CKD [28]. However, this study is relatively short in duration and small in sample size, and therefore does not provide meaningful information about the effect of pemafibrate on GFR. The JKAPI-CKD study will evaluate the effect of pemafibrate on kidney function using eGFR-cys or a subgroup analysis by the presence or absence of diabetes.

Recently, there has been increasing interest in TG-lowering therapy to prevent cardio-kidney events. A meta-analysis of 19 RCTs, including the PROMINENT trial, demonstrated that TG-lowering therapy was associated with a reduction in CVEs in patients with diabetes [4]. In a large national cohort study of US veterans with a long follow-up time, fibrate use was associated with a lower risk of ESKD and mortality [6]. A nested case-control study using data from a large administrative database also showed that fibrate use was associated with a reduced risk of MACE in patients with CKD [18]. Specifically, pemafibrate use, but not bezafibrate or fenofibrate use, was significantly associated with a decreased risk of MACE.

Several basic studies have reported on the mechanism underlying the renoprotective effects of pemafibrate. Pemafibrate ameliorated diabetic nephropathy through inhibition of kidney lipid content and oxidative stress in db/db mice [29]. A study using mice with fatty acid overload nephropathy demonstrated that pemafibrate attenuated tubular injury via modulating kidney fatty acid metabolism and inhibition of oxidative stress [30]. In addition, pemafibrate improved kidney inflammation and fibrosis in unilateral ureteral obstruction mice or adenine-induced CKD model mice [31, 32].

Thus, pemafibrate has been shown to be safely used in patients with CKD and may contribute to slowing CKD progression and reducing CV outcomes. The present study has several strengths, including the large sample size and long follow-up period. In addition, eGFR slope, which is the primary endpoint in the present study, was a reported surrogate marker for CKD progression in clinical trials [20, 21]. Furthermore, the large sample size allows for various subgroup analyses, which may lead to clinically meaningful findings. Notably, this study also has limitations. First, no clinical evidence will be obtained for CKD patients with eGFR <20 ml/min/1.73 m^2^ or for those undergoing maintenance dialysis. Second, the unblinded design may introduce bias. Third, the study population is expected to consist primarily of Japanese patients, which may limit the generalizability of the findings. Fourth, kidney function will be assessed using eGFR rather than measured GFR, and eGFR may be influenced by body composition, nutritional status, and severe proteinuria. However, sensitivity analyses excluding participants with conditions potentially affecting eGFR accuracy will be performed to evaluate the robustness of the results.

## CONCLUSION

The JKAPI-CKD study will evaluate the efficacy and safety of pemafibrate use in patients with hypertriglyceridemia complicated by CKD.

## Supplementary Material

sfag053_Supplemental_Files

## Data Availability

The datasets analyzed in the present study will be available from the corresponding author upon reasonable request.
