# Supplementary material for: Design of the Japan Kidney Association-Pemafibrate Intervention for Chronic Kidney Disease patients Study (JKAPI-CKD Study)
Source: Clin Kidney J. 2026 Feb 23;19(4):sfag053. doi: 10.1093/ckj/sfag053 (PMC13076029; doi:10.1093/ckj/sfag053)
Supplement: sfag053_Supplemental_Files [file sfag053_supplemental_files.zip › new_Supplementary Table 1_Visit schedule for observation, testing, and assessment_ver 2.docx]

| **Supplementary Table 1.** Visit schedule for observation, testing, and assessment. | | | | | | | | | | |
| --- | --- | --- | --- | --- | --- | --- | --- | --- | --- | --- |
| Items | Timing | | | | | | | | | |
|  | Eligibility confirmation | Consent Acquisition | Follow-up period | | | | | | | |
| Visit |  | | 1 | 2 | 3 | 4 | 5 | 6 | 7 |  |
| Week after treatment onset |  | within  −8 weeks | 0 | 12 | 26 | 52 | 78 | 104 | 8 weeks  after visit 6 | discontinuation |
| Allowance (weeks) | － | － | － | ±5 | ±8 | ±8 | ±8 | ±8 | ±4 | － |
| Informed consent | ● | | － | － | － | － | － | － | － | － |
| Eligibility confirmation | ● | － | － | － | － | － | － | － | － | － |
| Registration | － | ● |  |  |  |  |  |  |  |  |
| Characteristics | ● | － | － | － | － | － | － | － | － | － |
| Adherence to study treatment | － | － | － | ● | ● | ● | ● | ● | ○ | ○ |
| Status of concurrent therapy | ● | | ● | ● | ● | ● | ● | ● | ○ | ○ |
| Physical findings | － | | ● | ● | ● | ● | ● | ● | ○ | ○ |
| Blood analysis^#^ | ● | － | ● | ● | ● | ● | ● | ● | ○ | ○ |
| Urine analysis | － | | ● | ○ | ● | ● | ● | ● | ○ | ○ |
| Cardio-renal events | － | | ● | | | | | | ○ | ● |
| Adverse events | － | | ● | | | | | | ○ | ● |
| ●, required measurement; ○, optional measurement. #, eGFR, or serum creatinine, are collected optionally at the following times: −104 ±8 weeks, −52 ±8 weeks, −26 ±8 weeks, and −12 ±5 weeks before the study treatment onset. | | | | | | | | | | |
