# Supplementary material for: Design of the Japan Kidney Association-Pemafibrate Intervention for Chronic Kidney Disease patients Study (JKAPI-CKD Study)
Source: Clin Kidney J. 2026 Feb 23;19(4):sfag053. doi: 10.1093/ckj/sfag053 (PMC13076029; doi:10.1093/ckj/sfag053)
Supplement: sfag053_Supplemental_Files [file sfag053_supplemental_files.zip › new_Supplementary Table 2_Study Variables and Measurements._20251031.docx]

| **Supplementary Table 2. Study Variables and Measurements.** |
| --- |
| **Patient Characteristics at baseline** |
| Sex, Age |
| Cause of CKD: Diabetic nephropathy, Ischemic / Hypertensive nephrosclerosis, Glomerulonephritis (e.g., IgA nephropathy), Polycystic kidney disease, Post-kidney transplant, Other / Unknown |
| History and/or Presence of coronary artery disease, cerebrovascular disease, heart failure |
| Other Comorbidities / Medical History (Pulmonary disease, Hypertension, Diabetes Mellitus, Malignancy) |
| Alcohol Consumption / Smoking Status |
| **Assessment of study drug adherence** |
| Formulation, Dosage, and Adherence of Pemafibrate |
| **Verification of Concomitant Medications** |
| **Physical Examination** |
| At baseline: Height, Waist circumference  Body weight, Blood pressure (seated, resting), Heart rate  Calculated: Body mass index |
| **Laboratory Tests** |
| lipid metabolism |
| TG, HDL-C, total cholesterol, ^*^LDL-C (direct method)  Calculated^†^: LDL-C (calculated), non-HDL-C, TG/HDL-C ratio, small dense LDL-C, TG-rich lipoprotein cholesterol |
| Liver function |
| AST, ALT, γGTP, ALP |
| Kidney function |
| Creatinine, ^*^cystatin C  Calculated^†^: eGFR, ^*^eGFR-cys |
| Others |
| CK, BUN, urate, total protein, albumin, CRP,  RBC, WBC, platelet count, hemoglobin, hematocrit, blood glucose |
| Diabetes-specific collection^#^: Hemoglobin A1c |
| Heart Failure-specific collection^##^: BNP, or NT-proBNP |
| **Urinalysis (optional at Week 12; required at Weeks 0, 26, 52, 78, and 104)** |
| Urinary albumin (routine care only^¶^), Urine protein (UACR not tested^¶¶^), Urinary creatinine  Calculated: UACR, UPCR, Predicted ACR^¶¶¶^ |
| **Incidence of Cardio-Kidney Events^‖^** |
| **Assessment of Adverse Events** |
| *, optional measurement; ^#^, Collected only in participants with diabetes; **^##^**, Collected only in participants with heart failure; ^†^, Calculated using the formulas described in the Supplementary Methods (Supplementary File 2); ^¶^, with insurance coverage; ^¶¶^, assessed in patients without UACR measurement; ^¶¶¶^, predicted ACR = exp (5.2659 + 0.2934 × log (min (PCR/50, 1)) + 1.5643 × log (max (min (PCR/500, 1), 0.1)) + 1.1109 × log (max (PCR/500, 1)) − 0.0773 × (if female) + 0.0797 × (if diabetic) + 0.1265 × (if hypertensive). ^‖^, Kidney events: progression to ESKD (a sustained decline in eGFR to < 10 mL/min/1.73 m^2^), initiation of dialysis, kidney transplant, kidney-related death, or ≥ 40% eGFR decline from baseline. Cardiovascular events: cardiovascular death, non-fatal stroke, or non-fatal myocardial infarction. ACR: albumin-to-creatinine ratio; ALT: alanine aminotransferase; ALP: alkaline phosphatase; AST: aspartate aminotransferase; BNP: brain natriuretic peptide; BUN: blood urea nitrogen; CK: creatine kinase; CKD: chronic kidney disease; CRP: C-reactive protein; eGFR: estimated glomerular filtration rate; eGFR-cys: eGFR based on cystatin C; ESKD: end-stage kidney disease; γGTP: gamma-glutamyl transpeptidase; HDL-C: high-density lipoprotein cholesterol; LDL-C: low-density lipoprotein cholesterol; NT-proBNP: N-terminal pro BNP; RBC: red blood cell count; TG: triglyceride; UACR: urinary albumin-to-creatinine ratio; UPCR: urinary protein-to-creatinine ratio; WBC: white blood cell count. |
