# Supplementary material for: Design of the Japan Kidney Association-Pemafibrate Intervention for Chronic Kidney Disease patients Study (JKAPI-CKD Study)
Source: Clin Kidney J. 2026 Feb 23;19(4):sfag053. doi: 10.1093/ckj/sfag053 (PMC13076029; doi:10.1093/ckj/sfag053)
Supplement: sfag053_Supplemental_Files [file sfag053_supplemental_files.zip › new_Supplementary Table 3_Pre-specified categories for subgroup analyses_ver 4_20251031.docx]

| **Supplementary Table 3. Pre-specified categories for subgroup analyses.** |
| --- |
| **History of prior disease (presence vs. absence)** |
| Diabetes mellitus, hypertension, coronary artery disease, cerebrovascular disease, heart failure, pulmonary disease |
| **Cause of CKD** |
| Diabetic nephropathy, nephrosclerosis, glomerulonephritis, others or unknown |
| **Severity classification of CKD at baseline^#^** |
| eGFR: G3a, G3b, G4 |
| UACR or UPCR: A1, A2, A3 |
| **Laboratory values at baseline** |
| TG, LDL-C, CRP, AST, ALT, γ-GTP |
| **Serum TG level at 4 weeks** |
| **Presence or absence of concomitant medications** |
| Statins, EPA/DHA, RAS inhibitors (ACE-I, ARB, ARNI), SGLT2-inhibitors, MRA, HIF-Ph inhibitors, hyperuricemia medication |
| **Dose of pemafibrate** |
| **BMI category: <25, 25≤ <30, 30≤** |
| **Presence or absence of smoking** |
| **Presence or absence of drinking** |
| #, Severity classification of CKD is defined as follows: (G3a) 60 > eGFR ≥ 45 mL/min/1.73m^2^, (G3b) 45 > eGFR ≥ 30 mL/min/1.73m^2^, (G4) 30 > eGFR ≥ 15 mL/min/1.73m^2^, (A1) UACR < 30 mg/gCr, or UPCR < 0.15 g/gCr, (A2) 30 ≤ UACR < 300 mg/gCr, or 0.15 ≤ UPCR < 0.5 g/gCr, (A3) UACR ≥ 300 mg/gCr, or UPCR ≥ 0.5 g/gCr. ACE-I: angiotensin-converting-enzyme inhibitor; ALT: alanine aminotransferase; ARB: angiotensin II receptor blocker; ARNI: angiotensin receptor-neprilysin inhibitor; BMI: body mass index; AST: aspartate aminotransferase; CKD: chronic kidney disease; CRP: C-reactive protein; DHA: docosahexaenoic acid; eGFR: estimated glomerular filtration rate; EPA: eicosapentaenoic acid; γGTP: gamma-glutamyl transpeptidase; HIF-Ph: hypoxia-inducible factor prolyl-hydroxylase; LDL-C: low-density lipoprotein cholesterol; MRA: mineralocorticoid receptor antagonist; RAS: renin-angiotensin system; SGLT: sodium glucose cotransporter**;** TG: triglyceride; UACR: urinary albumin-to-creatinine ratio; UPCR: urinary protein-to-creatinine ratio. |
