# Supplementary material for: Design of the Japan Kidney Association-Pemafibrate Intervention for Chronic Kidney Disease patients Study (JKAPI-CKD Study)
Source: Clin Kidney J. 2026 Feb 23;19(4):sfag053. doi: 10.1093/ckj/sfag053 (PMC13076029; doi:10.1093/ckj/sfag053)
Supplement: sfag053_Supplemental_Files [file sfag053_supplemental_files.zip › Supplementary File 1_ORGANISATIONAL STRUCTURE of JKAPI-CKD study_ver 5_20251011.docx]

**Supplementary File 1. ORGANISATIONAL STRUCTURE of JKAPI-CKD study (as of August 1, 2025).**

**Research Organization:** Specified Nonprofit Corporation Japan Kidney Association (JKA) (chairman: Naoki Kashihara (Kawasaki Medical University)).

**Principal Investigator:** Kouichi Tamura (Yokohama City University).

**Vice Principal Investigator:** Yuichiro Yano (Juntendo University).

**Steering Committee:** Naoki Nakagawa (Asahikawa Medical University), Saori Nishio (Hokkaido University), Kouichi Asahi (Iwate Medical University), Kunihiro Yamagata (Tsukuba University), Akira Fukui (the Jikei University School of Medicine), Masaomi Nangaku (Tokyo University), Hirokazu Okada (Saitama Medical University), Ichiei Narita (Niigata Prefecture Health Promotion and Sports Medical Science Center, Niigata Prefecture Sports Association), Shoichi Maruyama (Nagoya University), Yoshitaka Isaka (Osaka University), Kazuhiko Tsuruya (Nara Medical University), Jun Wada (Okayama University), Yoshio Terada (Kochi University), Masashi Mukoyama (Omuta Tenryo Hospital), Enyu Imai (Nakayamadera Imai Clinic), Naoto Yokota (Yokota Clinic), and Kazuo Kobayashi (Internal Medicine Clinic Kobayashi).

**Director of Biostatistics:** Hiroshi Kanegae (Genki Plaza Medical Centre for Health Care).

**Clinical Design Advisor:** Hiddo Lambers Heerspink (Department of Clinical Pharmacology, University Medical Center Groningen).

**Contract Research Organization (CRO):** WDB COCO Corporation.

CRO is responsible for the research secretariat, data management, and data monitoring.

**Clinical Institutions and Principal Investigator of each institution:**

*Yokohama City University Hospital*: Koichi Tamura, *Okayama University Hospital*: Jun Wada, *Nagoya University Hospital*: Shoichi Maruyama, *Yokota Internal Medicine Clinic*: Naoto Yokota, *Kobayashi Internal Medicine Clinic*: Kazuo Kobayashi, *Kochi University Hospital*: Taro Horino, *Niigata University Medical and Dental Hospital*: Takashi Yamamoto, *Asahikawa Medical University Hospital*: Naoki Nakagawa, *Osaka University Hospital*: Yoshitaka Isaka, *The University of Tokyo Hospital*: Hiroshi Nishi, *Jikei University Hospital*: Yukio Maruyama, *The Jikei University Daisan Hospital*: Ai Katsuma, *Kawasaki Medical School Hospital*: Hajime Nagasu, *Iwate Medical University Hospital*: Kazuhiro Yoshikawa, *Iwate Medical University Uchimaru Medical Center*: Koichi Asahi, *Saitama Medical University Hospital*: Hirokazu Okada, *Kurume University Hospital*: Akimasa Taguchi, *Hiroshima University Hospital*: Takao Masaki, *Mie University Hospital*: Akira Katayama, *Tokai University Hospital*: Taemine Komaba, *University of Yamanashi Hospital*: Ayumu Nakajima, *Yokohama City University Medical Center*: Sho Kinguchi, *Hirosaki University Hospital*: Norio Nakamura, *Kagawa University Hospital*: Osamu Sobue, *Tokyo Medical University Hospital*: Yoshihito Moriyama, *Kanazawa University Hospital*: Takayoshi Iwata, *University of Tsukuba Hospital*: Kaori Mase, *Shinshu University Hospital*: Yuji Kamijo, *Shiga University of Medical Science Hospital*: Shinji Kume, *Kyoto University Hospital*: Motoko Yanagida, *Osaka Metropolitan University Hospital*: Masahiro Tsuda, *Shimane University Hospital*: Takeshi Kanda, *University of the Ryukyus Hospital*: Kentaro Kohagura, *Kobe University Hospital*: Hideki Fujii, *Iwatsuki Minami Hospital*: Yasuyuki Maruyama, *Tokyo Metropolitan Police Hospital*: Tomoya Okada, *Saiseikai Central Hospital*: Motoaki Komatsu, *Tottori University Hospital*: Tomoaki Takada, *Sasebo City General Hospital*: Yuki Ota, *Juntendo University Nerima Hospital*: Hiroaki Iio, *Juntendo University Urayasu Hospital*: Hitoshi Suzuki, *Oita University Hospital*: Hirotaka Shibata, *Beppu Medical Center*: Hidetoshi Kikuchi, *National Center for Global Health and Medicine Hospital*: Hideki Takano, *Nakatsu Daiichi Hospital*: Tomoko Nawata, *Kawashima Hospital*: Manabu Tashiro, *Agano City Hospital*: Kei Goto, *Ohta Internal Medicine Clinic*: Kazunari Ota, *Kyorin University Hospital*: Satoko Kawashima, *Kamei Hospital*: Hisato Shima, *Hirosaki Chuo Hospital*: Kenichi Shirato, *Ubukata Cardiovascular Clinic*: Satoshi Ubukata, *Kanazawa Medical University Hospital*: Kengo Furuichi, *Kumamoto University Hospital*: Takanari Kuwahara, *Koto Hospital*: Yuichi Tanaka, *Saga University Hospital*: Motoaki Miyazono, *Saiseikai Matsusaka General Hospital*: Eiji Ishikawa, *Ikeda Municipal Hospital*: Satoko Yamamoto, *Kagoshima University Hospital*: Yohito Yoshimine, *Teine Keijinkai Hospital*: Masato Moniwa, *Juntendo University Juntendo Tokyo Koto Geriatric Medical Center*: Hiroyuki Inoshita, *Kokura Memorial Hospital*: Hidetoshi Kanai, *Shinkawa Sumire Clinic*: Asako Matsuda, *Fukagawa Gatheria Clinic*: Takayuki Yokoyama, *Shizuoka General Hospital*: Kojiro Nagai, *Chiyoda Clinic*: Nobuhiko Sasaki, *Chiba University Hospital*: Katsuhiko Asanuma, *Otemae Hospital*: Toshio Sugiura, *Nakatsu Municipal Hospital*: Kohei Aoki, *Nagasaki University Hospital*: Tomoya Nishino, *Tottori Prefectural Central Hospital*: Chishio Munemura, *Oita Koseiren Tsurumi Hospital*: Makoto Arima, *Tokyo Women’s Medical University Hospital*: Hiroshi Kataoka, *Nisshin Orido Hospital*: Hiroshi Fujita, *Japan Sea General Hospital*: Takahiro Nakayama, *Fukui General Clinic*: Chie Yamamoto, *Yuri Kumiai General Hospital*: Masato Sawamura, *Ogachi Central Hospital*: Atsushi Komatsuda, *Yokosuka Kyosai Hospital*: Toshiharu Kokubo, *Yokosuka General Hospital Uwamachi*: Gaku Shimura, *Social Insurance Omuta Tenryo Hospital*: Masashi Mukoyama, *Gunma University Hospital*: Keiju Hiromura, *Nara Medical University Hospital*: Kazuhiko Tsuruya, *Seirei Sakura Citizen Hospital*: Takayuki Fujii, *Showa University Northern Yokohama Hospital*: Hirohito Sugawara, *Takinomiya General Hospital*: Yoko Nishijima, *Chikamori Hospital*: Kazunobu Yoshimura, *Hattori Hospital*: Shinichi Nishi, *Nagano Red Cross Hospital*: Mamoru Kobayashi, *National Hospital Organization Okayama Medical Center*: Kosuke Ota, *Juntendo University Hospital*: Takashi Kobayashi, *Nagahama City Hospital*: Yoshikata Morita, *Noshiro Kosei Medical Center*: Hajime Kaga, *Wakayama Medical University Hospital*: Shinichi Araki, *Shiomi Ekimae Internal Medicine Clinic*: Takanori Shimizu, *Ishikawa Prefectural Central Hospital*: Hiroshi Fujii, *Saitomo Soka Hospital*: Genki Goto, *Kasukabe Kisen Hospital*: Toshiharu Maruyama, *Dokkyo Medical University Saitama Medical Center*: Tetsuro Takeda, *Jichi Medical University Saitama Medical Center*: Haruhisa Miyazawa, *Omote Internal Medicine and Diabetes Clinic*: Keisuke Omote, *Tokyo Medical University Hachioji Medical Center*: Takashi Oda, *Kyorin University Suginami Hospital*: Miho Karube, *Omori Red Cross Hospital*: Ken Shibuya, *Saiseikai Yokohamashi Nanbu Hospital*: Sanae Saka, *Yokohama Medical Center*: Kei Matsushita, *Yokohama Sakae Kyosai Hospital*: Hitoshi Oshikawa, *Uonuma Kikan Hospital*: Noriaki Iino, *Saiseikai Niigata Hospital*: Yuya Sato, *Toyama Machinaka Hospital*: Maiko Ohara, *University of Fukui Hospital*: Nao Takahashi, *Fujita Memorial Hospital*: Ryoichi Miyazaki, *Matsunami General Hospital*: Takahiro Yajima, *Gifu Prefectural Tajimi Hospital*: Yutaka Sugiyama, *Agape Clinic*: Shimon Ito, *Fujita Health University Bantane Hospital*: Daiki Inaguma, *Okazaki City Hospital*: Hiroaki Asada, *Kasugai Municipal Hospital*: Yosuke Saka, *JA Aichi Koseiren Konan Kosei Hospital*: Tomohito Doke, *Fujita Health University Hospital*: Naoki Tsuboi, *Takeuchi Hospital*: Mika Fujimoto, *Otsu Municipal Hospital*: Jun Nakazawa, *Osaka Red Cross Hospital*: Kanenari Yahata, *Kitano Hospital, The Tazuke Kofukai Medical Research Institute*: Tatsuo Tsukamoto, *Okayama City Hospital*: Keiichi Takigami, *Kurashiki Central Hospital*: Noriaki Shimada, *Yamaguchi University Hospital*: Masaki Shibuya, *Minami-cho National Health Insurance Minami Hospital*: Soichi Honda, *Kyushu University Hospital*: Toshiaki Nakano, *Omuta City Hospital*: Makoto Nasu, *Oita Red Cross Hospital*: Eiji Uchida, *Imamura General Hospital*: Yoshiro Muraoka, *Kariyushi Clinic*: Ken Yamakawa, *Japan Community Health Care Organization Hokkaido Hospital*: Junya Yamamoto, *Kitami Kidney and Urology Clinic*: Nobuyuki Fukuzawa, *Yamagata University Hospital*: Issei Ichikawa, *Fukushima Medical University Hospital*: Kenichi Tanaka, *Omotesando Internal Medicine and Ophthalmology Clinic*: Tokuhiro Tsuchiya, *Matsuo-kai Clinic*: Yoshinori Ebihara, *Kenko-no-Mori Clinic*: Yataro Hosoda, *Nakagawa Clinic*: Haruo Nakagawa, *Tokorozawa Hakushokai Hospital*: Toshihiko Imakiire, *Ogawa Cardiovascular Internal Medicine Clinic*: Takanori Ogawa, *Yamaki Internal Medicine Clinic*: Mariro Yamaki, *Maruyama Memorial General Hospital*: Akio Maeda, *Seino Internal Medicine Clinic*: Hiroaki Seino, *Hyogo College of Medicine Hospital*: Takahiro Kuragano, *Minamiuonuma City Hospital*: Kaoru Tabei, *Masuda Hospital*: Fumiko Kudo, *Ami-Higashi Clinic*: Tetsuya Kasuga, *Miura Central Clinic*: Masahiro Takibata, *Kita 5-jodori Internal Medicine and Cardiology Clinic*: Masanao Naya, *Nakata Clinic*: Shinsuke Nakata, *Gyotoku General Hospital*: Akira Murasawa, *Tsubokawa Internal Medicine Clinic*: Toshinari Tsubokawa, *Yodakubo Hospital*: Satoshi Shiroshita, *Shiota Hospital*: Koichi Nomura, *Urayasu Tsubame Clinic*: Masahiro Sakai, *Nerima Hikarigaoka Hospital*: Hideyuki Okuma, *Inagaki Heart Clinic*: Koichi Inagaki, *Asama General Hospital*: Eita Nishimori, *Minamino Cardiovascular Hospital*: Yoshiki Hata, *Higashi-Ojima Medical Clinic*: Munehiko Shibata, *Hotaruno Central Internal Medicine Clinic*: Manabu Uchida, *Murase Hospital*: Akiko Tanoue, *Takamatsu Municipal Minna-no Hospital*: Naoki Muguruma, *Shimizu Clinic*: Kiyoyasu Shimizu, *Machino Internal Medicine Clinic*: Hiroyuki Machino, *Tsuboi Clinic*: Shuta Tsuboi, *Katsuya Clinic*: Tomohiro Katsuya, *Niigata Shirone General Hospital*: Koji Matsuo, *Tokushima Prefectural Miyoshi Hospital*: Norihito Kageyama, *Hashimoto Kidney Clinic*: Masami Hashimoto, *Shirakawa Kosei General Hospital*: Tsuyoshi Iwasaki, *Tanaka Clinic*: Seiichi Tanaka, *Tokyo Station Center Building Clinic*: Arihiro Kiyosue, *Hokkaido Cancer Center*: Hitoki Inoue, *Aomori Prefectural Central Hospital*: Masamichi Nakata, *Kumanomae Nishimura Internal Medicine Clinic*: Hideki Nishimura, *Okuda Clinic*: Takeshi Okuda, *Takada Clinic*: Yoshihisa Takada, *Kaneko Internal Medicine and Cardiology Clinic*: Akihiro Kaneko, *Yokota Internal Medicine Clinic*: Kazuki Yokota, *Nakada Internal Medicine Clinic*: Nobuhiro Omura, *Okazaki Heart Clinic*: Osamu Okazaki, *Hiroshima Prefectural Hospital*: Toshinori Ueno, *Hosoya Clinic*: Tsuyoshi Hosoya, *Kagoshima City Hospital*: Yozo Yoshimine, *Koyama East Clinic*: Kunihiro Suzuki, *Tamachi Clinic*: Tsuguto Abe, *Isahaya General Hospital, Japan Community Health Care Organization*: Ryosuke Sakamoto, *Oita Prefectural Hospital*: Naoya Fukunaga, *Iitake Internal Medicine Clinic*: Chie Iitake, *Kikuma Clinic*: Kenji Yamauchi, *Kimitsu Central Hospital*: Masashi Aizawa, *Shirokane Takanawa Ekimae Internal Medicine and Diabetes Clinic*: Eisuke Yasunari, *Akutsu Clinic*: Takashi Anno, *Sainokuni Clinic*: Yasuhiro Ohara, *Kashiwabara Clinic*: Kyohei Yamazaki, *Hishiki Clinic*: Toshimasa Hishiki, *Fukuyama City Hospital*: Akifumi Onishi, *Yokohama Hodogaya Central Hospital*: Shinichiro Yoshida, *Hadano Station South Exit Clinic*: Takamoto Furuki, *Ryuo Mitsui Clinic*: Fumihiko Mitsui, *Kobe City Medical Center General Hospital*: Akihiro Yoshimoto, *Nakamurabashi Ekimae Internal Medicine Clinic*: Akimasa Shinohara, *Umeda Internal Medicine Clinic*: Jun Umeda, *Chiba Clinic*: Hideo Chiba, *Tokushima University Hospital*: Osamu Wakino, *Shin-Matsudo Central General Hospital*: Eiichi Sato, *Odawara Municipal Hospital*: Keisuke Soeda, *Akatsuka Clinic*: Gen Akatsuka, *Sumita Internal Medicine Clinic*: Koichi Sumita, *Gamagori Clinic*: Kazutaka Murakami, *Sengawa Kidney and Rheumatology/Internal Medicine Clinic*: Shintaro Masuko, *Yamato Municipal Hospital*: Yasuyo Takeshita, *Nippon Life Hospital*: Takashi Uzu, *Komizo Clinic*: Yoshimi Komizo, *Katsuragawa Saito Internal Medicine Clinic*: Nariyuki Saito, *Haraguchi Internal Medicine and Kidney Clinic*: Kazutaka Haraguchi, *Oseto Internal Medicine Clinic*: Susumu Oseto, *Magome Internal Medicine and Kidney Clinic*: Teiko Inoue, *Naka Memorial Clinic*: Ken Osinoi, *Yamane Hospital*: Yuko Yamane, *Teine Nephrology Clinic*: Hiroya Mukai, *Otsu Red Cross Hospital*: Toshiyuki Furumiya, *Tsuchiura Beryl Clinic*: Homare Shimohata, *Miyazaki Clinic*: Mitsuhiro Miyazaki, *Sengawa Hiro Clinic*: Hiroshi Suzuki, *Ikejiri Internal Medicine and Surgery Clinic*: Reiko Ikejiri, *Yoshida Hospital, Keiyu-kai Medical Corporation*: Mizuho Okada, *Kurihara Internal Medicine Clinic*: Hiroyoshi Kurihara, *Nagoya Memorial Hospital*: Miho Tatematsu, *Osaka General Medical Center*: Yoshiyasu Ueda, *Well-being Internal Medicine Clinic*: Naohiro Nomura, *Motoshima General Hospital*: Toshio Kawada, *Tokushima Prefectural Central Hospital*: Ryoichi Nakanishi, *Fujisawa City Hospital*: Masashi Sakai, *Clinic Yokoyama*: Toshio Yokoyama, *Nakatsukasa Adachi Clinic*: Katsuhiko Nakatsukasa, *Matsuda Gastroenterology and Diabetes Clinic*: Fumihiro Matsuda, *Yokoyama Internal Medicine and Pediatrics Clinic*: Takanori Yokoyama, *Ando Internal Medicine Clinic*: Tatsuya Ando, *Yokohama Rosai Hospital*: Kazuki Watanabe, *Okada Clinic*: Yoshiharu Okada, *Atsugi City Hospital*: Hideo Okonogi, *Matsuo Kenko Clinic*: Kaneyuki Matsuo, *Osugi Internal Medicine Clinic*: Yukio Osugi, *Hattori Clinic II*: Takamasa Miyauchi, *Tanita Ikeda Clinic*: Jo Nagakura, *Nagasaki Hospital, Medical Corporation*: Manabu Takahashi, *Fukui-ken Saiseikai Hospital*: Yasutaka Kamikawa, *Shinkoiwa Internal Medicine Clinic*: Satoshi Kawakami, *Kenwakai Hospital*: Yuta Hara, *Arao City Ariake Medical Center*: Yusuke Hata, *Tokushima Municipal Hospital*: Tomoharu Fukumori, *Kuwana City Medical Center*: Masashi Yasutomi, *Sayama General Clinic*: Shiko Moto, *Sayama Kidney Clinic*: Naofumi Ikeda, *Kinashi Obayashi Hospital*: Hiroaki Obayashi, *Yurakubashi Clinic*: Toshiyuki Hayashi, *Sawada Hepatology and Gastroenterology Clinic*: Yukio Sawada, *Toto Kasukabe Hospital*: Miwa Bando, *Nakada Clinic*: Kuniya Nakada, *Osaka Saiseikai Senri Hospital*: Noriyo Kubo, *Taniguchi Clinic*: Takahiro Taniguchi, *Arai Internal Medicine Clinic*: Yoshiyuki Arai, *Otsuka Kitaguchi Clinic*: Hiromitsu Sato, *Nagusa Clinic*: Mitsunobu Hibino, *Meisei Hospital*: Nobuhiko Takahara, *My Clinic Araki*: Motoharu Araki, *Asano Internal Medicine Clinic*: Shogo Asano, *Toride Nephrology and Urology Clinic*: Yukihide Matsui, *Chukyo Clinical*: Taku Komori, *Kitamura Clinic*: Taketoshi Kitamura, *Ebino Centro Clinic*: Akira Nagai, *Dokkyo Medical University Nikko Medical Center*: Takanori An, *Tansho Internal Medicine, Nephrology, Dermatology Clinic*: Kosuke Tansho, *Nakadori General Hospital*: Daisuke Matsuda, *Hashimoto Internal Medicine, Diabetes, and Endocrinology Clinic*: Kenichi Hashimoto, *Okachimachi Ohisama Internal Medicine Clinic*: Kenichiro Enokio, *Mizuno Clinic, Hosuikai Medical Corporation*: Masao Mizuno, *Ogami Internal Medicine and Pediatrics Clinic*: Hiroki Ogami, *Higashikori Hospital*: Satoshi Mikami, *Ishikura Internal Medicine Clinic*: Kazuhide Ishikura, *Yabuki Hospital*: Ikuto Masakane, *Ageo Heart Clinic*: Tetsutaro Shimaoka, *Mikawashima Tower Clinic*: Kazumitsu Omori, *Kyoto Medical Center*: Koji Takaori, *Yokohama Minami Kyosai Hospital*: Daisuke Kanai, *Kyushu Central Hospital*: Koji Mansei, *Tokuyama Central Hospital*: Tatsuyo Takahashi, *Takeda General Hospital*: Keiichiro Nakamae, *Gifu University Hospital*: Yoshinari Yasuda, *Itabashi Chuo General Hospital*: Shuzo Kaneko, *Matsumoto Clinic, Shokokai Medical Corporation*: Kazutaka Matsumoto, *Yutenji Internal Medicine Clinic*: Kotaro Shimokawa, *Sato Juichi Clinic*: Juichi Sato, *Kure Medical Center*: Shunsuke Takahashi, *Soyama Clinic*: Nobuhiko Soyama, *Odate Municipal General Hospital*: Susumu Ikeshima, *Gifu Prefectural General Medical Center*: Ichiro Murata, *Ichiriyama Imai Hospital*: Fumihiro Imai, *Ichinoe Ekimae Himawari Clinic*: Daisuke Ito, *Mashiko Hospital, Kenjin-kai Medical Corporation*: Shogo Shimizu, *Seiwadai Clinic*: Sayaka Horii, *Hitokuchizaka Clinic*: Akiko Sakai, *Toyama University Hospital*: Tsutomu Koike, *Okyoduka Clinic*: Toshiki Tatsumura, *Kataoka Clinic*: Hideki Kataoka, *Osumi Kanoya Hospital*: Yukihiro Tamura, *Nerima General Hospital*: Koichiro Azuma, *Nara Prefectural General Medical Center*: Hideo Tsushima, *Minami Osaka Hospital*: Yasuro Kumeda, *Daido Clinic*: Hideaki Shimizu, *Nonoichi Medical Clinic*: Shigeru Nakano, *Koike Clinic*: Seigo Kurisu, *Japanese Red Cross Society Wakayama Medical Center*: Shota Komidori, *Tokyo Medical University Ibaraki Medical Center*: Koichi Hirayama, *Toho University Ohashi Medical Center*: Nobuhiko Tsuneki, *Matsubara-kai Matsuyama Daiichi Hospital*: Fumiaki Matsubara, *Ozeki Clinic*: Norishige Ozeki, *Kitami Red Cross Hospital*: Takahiro Nagashima, *Hokkaido University Hospital*: Saori Nishio, *Hamamatsu Medical Center*: Takayuki Tsuji, *Tsumura Cardiovascular Clinic*: Yasuhiro Tsumura, *Ishida Clinic*: Ryo Ishida, *Shinzawa Surgery Clinic*: Toshimitsu Shinzawa, *Kyoto First Red Cross Hospital*: Toru Tanaka, *Kanazawa Medical University Himi Municipal Hospital*: Toshihiro Higashikawa, *Minami Koshigaya Kenshinkai Clinic*: Yuki Shuto, *Kawaguchi Medical Clinic*: Mitsuhiko Kawaguchi, *Clinic Hibiaoao*: Kazuya Takazawa, *Takeda Clinic, Medical Corporation*: Takako Yamamoto, *Dan Clinic*: Nobuhiro Dan, *Bunkabashi Family Clinic*: Hideki Kurihara, *Nagasawa Clinic*: Arata Nagasawa, *Hikone Municipal Hospital*: Akira Kuroe, *Kurose Clinic*: Kikuo Kurose, *Tohoku Rosai Hospital*: Manabu Kanda, *Kyobashi Lifestyle and Heart Clinic*: Hiroki Uehara, *Diabetes and Endocrinology Internal Medicine Clinic Tosaki*: Takahiro Tosaki, *Sumitomo Besshi Hospital*: Naoya Horimoto, *Miyazaki RC Clinic*: Masaki Miyazaki, *Tokutake Clinic*: Eiichi Tokutake, *Sakamoto Internal Medicine Clinic*: Yasuaki Sakamoto, *Tanaka Internal Medicine Clinic*: Yuya Tanaka, *Sapporo Fushiko Internal Medicine and Dialysis Clinic*: Masataka Kakuta, *Asahi University Hospital*: Junichiro Yamamoto, *Kagayaki Internal Medicine and Diabetes Clinic*: Tsuyoshi Harada, *Nakajima Diabetes Internal Medicine Clinic*: Yoshiki Nakajima, *Miyamoto Internal Medicine Clinic*: Takahide Miyamoto, *Misato Central General Hospital*: Yoshiyuki Yazaki, *Uenoue Ueda Clinic*: Ryoichi Ueda, *Sawada Clinic*: Makoto Sawada, *Doi Internal Medicine Clinic*: Takahiro Doi, *Maebashi Hirosegawa Clinic*: Shintaro Yano, *Yoshiyama Clinic*: Izumi Yoshiyama, *Sakura Internal Medicine and Diabetes Clinic*: Hiroshi Awasaki, *Matsutani Internal Medicine Clinic*: Norihiko Matsutani, *Nakajima Internal Medicine Clinic*: Yasushi Nakajima, *Kansai Electric Power Hospital*: Akira Ishii, *Showa University Fujigaoka Hospital*: Marie Ito, *Kokubu Clinic*: Kazushi Kokubu, *IMS Tokyo Katsushika General Hospital*: Takayuki Sunohara, *Kenritsu Futaba-no-Sato Hospital*: Hiroki Terakawa, *OBP Imamura Clinic*: Shuzo Imamura, *Aomori Jikeikai Hospital*: Kota Sasaki, *Oshima Internal Medicine and Dermatology Clinic*: Yasushi Oshima, *Kumatori Sakaguchi Clinic*: Yasuhiro Sakaguchi, *Saitama City Hospital*: Sanren Iwashita, *Tanaka Internal Medicine Clinic*: Yuta Inoue, *Nabatake Fuyuno Clinic*: Seiya Fuyuno, *Nishina Internal Medicine and Diabetes/Endocrinology Clinic*: Shuhei Nishina, *Fukaya Red Cross Hospital*: Noriaki Henmi, *Fukuoka University Hospital*: Kosuke Masutani, *Fujiki Internal Medicine and Surgery Clinic*: Rei Fujiki, *Mooca Internal Medicine and Diabetes Clinic*: Kazunori Yanagi, *Ryoshukai Fujii Hospital, Kidney Disease Research Institute*: Masatoshi So, *Ehime Prefectural Central Hospital*: Taichi Murakami, *Idamae Internal Medicine Clinic*: Masamichi Yamada, *Ayame Internal Medicine Clinic*: Hideo Ayame, *Kameda Daiichi Hospital, Aijinkai Medical Corporation, Gastrointestinal Endoscopy Center*: Azuma Watanabe, *Imamura Clinic*: Minoru Imamura, *Iwai Clinic*: Toshio Iwai, *Harada Hospital, Ichiyokai Medical Corporation*: Toshiki Doi, *Tatsu Clinic*: Yoshinobu Sato, *Utsunomiya Memorial Hospital, Head of Kidney and Dialysis Center, Head of Kidney Surgery Department*: Koji Namiki, *Okabe Hospital*: Tsuyoshi Horikawa, *Kasai Shoikai Hospital*: Yasuhiro Kuroi, *Chigasaki Municipal Hospital*: Shinichiro Masuda, *Kijima Hospital Hon-in*: Hideyuki Kijima, *Yoshinogawa Medical Center, Kidney Center*: Hideki Hayashi, *Kyoto Okamoto Memorial Hospital*: Akio Kishi, *Kyoto Second Red Cross Hospital*: Masahiro Yamazaki, *Kyoto Prefectural University of Medicine Hospital*: Keiichi Tamagaki, *Kuwana Medical Clinic*: Yasuhiro Hotta, *Public Koka Hospital*: Naoko Takeda, *National Institute of Health Crisis Management, National Kokubunai Medical Center*: Nobuyuki Katsuyama, *Saga Prefectural Medical Center Koseikan*: Megumi Nakamura, *Saiseikai Kanagawa Prefectural Hospital*: Kuniki Usui, *Nara City Hospital*: Yoshiharu Nishitani, *Doai Memorial Hospital*: Yu Yamaguchi, *Shibukawa Medical Center*: Junshi Masada, *Konuma Internal Medicine and Gastroenterology Clinic*: Hironori Konuma, *Oda Internal Medicine Clinic*: Hiroaki Oda, *Ono Internal Medicine Clinic*: Toru Sakairi, *Akishima Rheumatology and Collagen Disease Internal Medicine Clinic*: Takuya Yoshioka, *Showa University Koto Toyosu Hospital*: Kei Matsumoto, *Matsumoto Clinic*: Hiroaki Matsumoto, *Shonan Kamakura General Hospital, Comprehensive Kidney Disease Medical Center*: Hisami Hidaka, *Chita Peninsula Rinku Hospital*: Ryo Tomita, *Fukamizu Clinic*: Ryo Fukamizu, *Fukaya Clinic*: Reona Fujii, *Kobe Rosai Hospital*: Minoru Sato, *Kanda Ekihigashi-guchi Clinic*: Kosuke Mabuchi, *Jinken Clinic*: Yudai Isozaki, *Mizuno Memorial Hospital*: Isamu Yokoe, *Seishin-kita Clinic*: Takeshi Ono, *Sendai Yanagiu Clinic*: Taro Fukushi, *Funabashi Ekimae Internal Medicine Clinic*: Akiyo Masumura, *Osaka City General Hospital*: Daisuke Yamasaki, *Osaki Municipal Hospital*: Yoichiro Chikamatsu, *Takenotsuka Diabetes and Dermatology Clinic*: Genritsu Sato, *Tokyo Medical Center*: Tomokazu Matsuura, *Tokyo Dental College Ichikawa General Hospital*: Hirofumi Tokuyama, *Tokyo Women’s Medical University Adachi Medical Center*: Tetsuya Ogawa, *Tokyo Metropolitan Hiroo Hospital*: Masato Tajima, *Toho University Sakura Medical Center*: Yasushi Ohashi, *Nishizume Clinic, Diabetes Department*: Suzuko Iwami, *Nara Prefectural Seiwa Medical Center*: Katsuhiko Morimoto, *Uchiyama Clinic*: Kazuaki Uchiyama, *Minami Aoyama Internal Medicine Clinic*: Takako Suzuki, *Nihonbashi Kawamata Internal Medicine Clinic*: Hirofumi Kawamata, *Hakuai Hospital, Dialysis Center*: Masami Nagasue, *Hatchobori Ishikawa Internal Medicine Clinic*: Yuji Ishikawa, *Iwata City Hospital*: Hirotaka Fukazawa, *Toyama City Hospital*: Satoshi Ota, *Hiramatsu Clinic*: Soichiro Hiramatsu, *Kitajimabashi Clinic*: Junichi Ueda, *Akashi Medical Center*: Yuriko Yonekura, *Meihou Clinic*: Akio Kimura, *Dokkyo Medical University Hospital*: Shigeru Toyoda, *Ehime University Hospital*: Yoichi Hiasa, *Aichi Medical University Hospital*: Takuji Ishimoto, *Saitama Medical University General Medical Center*: Hajime Hasegawa, *Jinai Clinic*: Nakayuki Yoshimura, *Matsushita Memorial Hospital*: Kazuhiro Sonomura, *Solis Sapporo Clinic*: Ichiro Sakuma, *Iwamoto Clinic*: Yasuto Iwamoto, *Kawaguchi City Medical Center*: Yasushi Kanazawa, *Sakura Memorial Hospital*: Akira Kurosawa, *Saitama Medical University Kawagoe Clinic*: Mariko Sato, *Tokyo Bay Urayasu Ichikawa Medical Center*: Toshihiko Suzuki, *Sourage Internal Medicine Clinic*: Motoichi Iwahori, *International University of Health and Welfare Narita Hospital*: Kiyotaka Uchiyama, *Japan Community Health Care Organization Osaka Hospital*: Akira Suzuki, *Kanda Nishiguchi Uchida Internal Medicine Clinic*: Toshiya Uchida, *Kagurazaka Clinic*: Tohru Abe, *Suzuki Clinic*: Maiko Suzuki, *Tamaki Clinic, Shoyo-kai Medical Corporation*: Noboru Tamaoki, *Sakakibara Clinic*: Eiji Sakakibara, *Niigata Diabetes Clinic*: Katsunori Suzuki, *Yoshioka Internal Medicine Clinic*: Taishi Yoshioka, *Gosen Rokutou Clinic*: Katsuya Kimura, *Shinrakuen Hospital*: Makoto Goto, *Shoseikai Toyama Hospital*: Akihiro Nimura, *Takaoka Minami Heart Center Minami-no-mori Hospital*: Munenori Ota, *Takaoka City Hospital*: Takuya Nakahashi, *Adachi Kidney, Dialysis, and Hypertension Clinic*: Hiroki Adachi, *Sugita Genpaku Memorial Obama Municipal Hospital*: Haruyoshi Yoshida, *Yamaura Internal Medicine Clinic*: Shuichi Yamaura, *Hamamatsu University Hospital*: Hideo Yasuda, *Juntendo University Shizuoka Hospital*: Yoshio Shimizu, *Sasaki Heart Clinic*: Akitoshi Sasaki, *Nagoya Dai-ni Red Cross Hospital, Japanese Red Cross Society Aichi Medical Center*: Shoji Saito, *Nagoya Dai-ichi Red Cross Hospital, Japanese Red Cross Society Aichi Medical Center*: Kaori Yasuda, *Kyoto Tanabe Central Hospital, Artificial Dialysis Department*: Nobuhisa Hagiwara, *Minamitani Clinic, Minamitani Keifukai Medical Corporation*: Naoto Minamitani, *Kaizuka Nishide Clinic*: Osamu Nishikawa, *Sakaguchi Clinic*: Masayoshi Sakaguchi, *Moriyama Clinic*: Yasunari Moriyama, *Maeda Clinic*: Yasuhiro Maeda, *Someya Clinic*: Yoshihisa Kinoshita, *Sano Internal Medicine Clinic*: Tetsuaki Sano, *Yamaguchi Saiseikai Shimonoseki General Hospital*: Yutaka Nitta, *Kagawa Prefectural Central Hospital*: Hiroyuki Watatani, *Kurume General Hospital*: Takuma Hazama, *Nagae Clinic*: Kanji Ota, *Tetsuo Internal Medicine Clinic*: Hirokuni Tetsuo, *Nishiura Hospital*: Ryosuke Nishiura, *Green Bird Clinic*: Kazuhiro Yoshimoto, *Miyaji Clinic*: Noriaki Miyaji, *Yamaguchi Hiroshi Clinic*: Hiroshi Yamaguchi, *Kaiho Hospital*: Ryo Tominagoe, *Shinkenkou Clinic*: Masato Oroku, *Japan Community Health Care Organization Saitama Hokubu Medical Center*: Minami Toda
