# Supplementary material for: Design of the Japan Kidney Association-Pemafibrate Intervention for Chronic Kidney Disease patients Study (JKAPI-CKD Study)
Source: Clin Kidney J. 2026 Feb 23;19(4):sfag053. doi: 10.1093/ckj/sfag053 (PMC13076029; doi:10.1093/ckj/sfag053)
Supplement: sfag053_Supplemental_Files [file sfag053_supplemental_files.zip › Supplementary File 2_Formulas Used for Lipid and Renal Function Calculations_20250924.docx]

**Supplementary File 2. Formulas Used for Lipid and Renal Function Calculations.**

**Lipid metabolism**

- LDL-C = TC – HDL-C – TG/5 (Friedewald formula [1])
- sdLDL-C = LDL-C* – (1.43 × LDL-C* – 0.14 × [In(TG) × LDL-C*] – 8.99) [2]

(*LDL-C is calculated using the Sampson formula [3]; LDL-C = TC/0.948 – HDL-C/0.971 – [TG/8.56 + (TG × non-HDL-C)/2140 – TG^2^/16100] – 9.44)

- TRL-C = TC – LDL-C (direct method) – HDL-C [4]

**Renal Function**

- eGFR (mL/min/1.73m^2^) = 194 × Cr^–1.094^ × Age^–0.287^ (for male) [5]
- eGFR (mL/min/1.73m^2^) = 194 × Cr^–1.094^ × Age^–0.287^ × 0.739 (for female) [5]
- eGFR-cys (mL/min/1.73m^2^) = {104 × CysC^–1.019^ × 0.996 ^Age^} – 8 (for male) [6]
- eGFR-cys (mL/min/1.73m^2^) = {104 × CysC^–1.019^ × 0.996 ^Age^ × 0.929} – 8 (for female) [6]

**Reference**

1. Friedewald WT, Levy RI, Fredrickson DS. Estimation of the concentration of low-density lipoprotein cholesterol in plasma, without use of the preparative ultracentrifuge. Clin Chem. 1972; 18: 499-502.
2. Sampson M, Wolska A, Warnick R, et al. A New Equation Based on the Standard Lipid Panel for Calculating Small Dense Low-Density Lipoprotein-Cholesterol and Its Use as a Risk-Enhancer Test. Clin Chem. 2021; 67: 987-997.
3. Sampson M, Ling C, Sun Q, et al. A New Equation for Calculation of Low-Density Lipoprotein Cholesterol in Patients With Normolipidemia and/or Hypertriglyceridemia. JAMA Cardiol. 2020; 5: 540-548.
4. Hermans MP, Ahn SA, Rousseau MF. Novel unbiased equations to calculate triglyceride-rich lipoprotein cholesterol from routine non-fasting lipids. Cardiovasc Diabetol. 2014; 13: 56.
5. Matsuo S, Imai E, Horio M, et al; Collaborators developing the Japanese equation for estimated GFR. Revised equations for estimated GFR from serum creatinine in Japan. Am J Kidney Dis. 2009; 53: 982-92.
6. Horio M, Imai E, Yasuda Y, et al; Collaborators Developing the Japanese Equation for Estimated GFR. GFR estimation using standardized serum cystatin C in Japan. Am J Kidney Dis. 2013; 61: 197-203.
